# Breast cancer metastasis suppressor 1 (BRMS1) inhibits osteopontin transcription by abrogating NF-κB activation

**DOI:** 10.1186/1476-4598-6-6

**Published:** 2007-01-16

**Authors:** Rajeev S Samant, David W Clark, Rebecca A Fillmore, Muzaffer Cicek, Brandon J Metge, Kondethimmana H Chandramouli, Ann F Chambers, Graham Casey, Danny R Welch, Lalita A Shevde

**Affiliations:** 1 Mitchell Cancer Institute, University of South Alabama, Mobile, Alabama, USA; 2 Lerner Research Institute, Cleveland Clinic Lerner School of Medicine, Ohio, USA; 3 Department of Cancer Biology, The London Regional Cancer Program, London, Ontario, Canada; 4 Department of Pathology, The University of Alabama at Birmingham, Birmingham, Alabama, USA; 5 Cell Biology and Pharmacology/Toxicology, The University of Alabama at Birmingham, Birmingham, Alabama, USA; 6 Comprehensive Cancer Center, The University of Alabama at Birmingham, Birmingham, Alabama, USA; 7 National Foundation for Cancer Research-Center for Metastasis Research, The University of Alabama at Birmingham, Birmingham, Alabama, USA

## Abstract

**Background:**

Osteopontin (OPN), a secreted phosphoglycoprotein, has been strongly associated with tumor progression and aggressive cancers. MDA-MB-435 cells secrete very high levels of OPN. However metastasis-suppressed MDA-MB-435 cells, which were transfected with breast cancer metastasis suppressor 1 (BRMS1), expressed significantly less OPN. BRMS1 is a member of mSin3-HDAC transcription co-repressor complex and has been shown to suppress the metastasis of breast cancer and melanoma cells in animal models. Hence we hypothesized that BRMS1 regulates OPN expression.

**Results:**

The search for a BRMS1-regulated site on the OPN promoter, using luciferase reporter assays of the promoter deletions, identified a novel NF-κB site (OPN/NF-κB). Electrophoretic mobility shift assays and chromatin immunoprecipitations (ChIP) confirmed this site to be an NF-κB-binding site. We also show a role of HDAC3 in suppression of OPN *via *OPN/NF-κB.

**Conclusion:**

Our results show that BRMS1 regulates OPN transcription by abrogating NF-κB activation. Thus, we identify OPN, a tumor-metastasis activator, as a crucial downstream target of BRMS1. Suppression of OPN may be one of the possible underlying mechanisms of BRMS1-dependent suppression of tumor metastasis.

## Background

Several reports have linked the phosphoglycoprotein, osteopontin (OPN), with tumor progression and metastatic spread [1-4]. The levels of OPN are significantly elevated in the tumors and plasma of patients with metastatic breast cancer and are notably associated with tumor aggressiveness and decreased patient survival [1,2]. Alterations in the gene expression profile upon OPN over-expression in a breast cancer cell line include changes in genes classically associated with early stages of tumor formation [5]. We have recently found that OPN contributes to early breast tumor development [6].

Breast cancer metastasis suppressor 1 (BRMS1) has been shown to suppress metastasis of metastatic human breast cancer and melanoma lines as well as mouse mammary cancers [7-9]. BRMS1 is a member of the mSin3-HDAC transcription co-repressor complex [10]. This finding suggests that BRMS1 may modulate expression of other genes. Hence, we were interested in investigating the downstream signaling modulated by BRMS1. We present evidence that expression of tumor/metastasis promoting OPN is downregulated by BRMS1. We describe a novel NF-κB site in the OPN promoter and show that BRMS1 interferes with the activation of NF-κB leading to the suppression of OPN. We also show a role of HDAC3 in suppression of OPN *via *OPN/NF-κB.

## Results

### BRMS1 suppresses OPN expression

**Figure 1:**
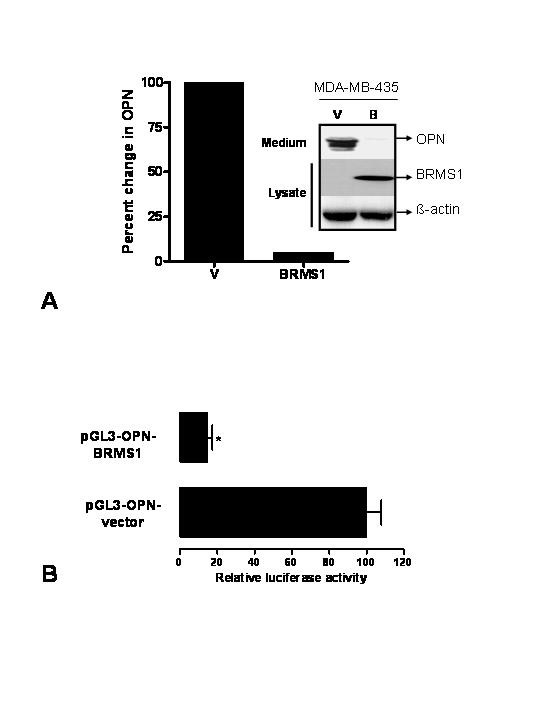
**A**. Expression of OPN is down-regulated by 95% in the 435/BRMS1 cells. Serum-free conditioned medium from equal numbers of pcDNA transfected MDA-MB-435 (V) and 901-BRMS1-transfected 435 cells, 435/BRMS1 (BRMS1) was resolved using a 12.5% SDS-PAGE, transferred to a PVDF membrane and probed with the anti-human OPN monoclonal antibody [30]. The bar graph depicts the percent change in OPN expression based on densitometric analysis of the immunoblot. To confirm the BRMS1 expression in these cells, the cells were lysed in NP-40 lysis buffer and 30 ug of protein was resolved using a 12.5% SDS-PAGE, transferred to a PVDF membrane and probed with the anti-901 monoclonal antibody (for BRMS1 epitope). The membrane was reprobed for levels of β-actin to confirm equal loading. **B**. BRMS1 suppresses activity of the human OPN promoter. COS-7 cells were co-transfected with pGL3-OPN [28] and pCMV-myc or pCMV-myc-BRMS1 using Lipofectamine 2000 (Invitrogen). Luciferase activity was normalized to the total protein concentration. Data is expressed as Relative luciferase activity, where control is 100%. The data represents five independent experiments in triplicate. * indicates significant suppression (p < 0.05).

Restoration of BRMS1 expression in the metastatic breast cancer cell line MDA-MB-435 (435) suppresses its metastatic capability in nude mice [7]. Gene expression analysis revealed that the expression of OPN was down-regulated by 95% in the 435/BRMS1 cells compared to vector-transfected 435 cells. We confirmed this observation by an immunoblot analysis of the conditioned cell-free, serum-free supernatant from the two cell lines. We saw a dramatic reduction in the level of secreted OPN from 435/BRMS1 cells (Fig. 1A) as well as decreased expression of OPN in the cell lysate (data not shown). Consistent with these findings, we saw that the 435/BRMS1 cells were only marginally (15–20% of the control) able to activate the luciferase reporter of OPN promoter (Fig. 1B).

### The OPN promoter construct lacking the NF-κB site is relieved for suppression by BRMS1

**Figure 2:**
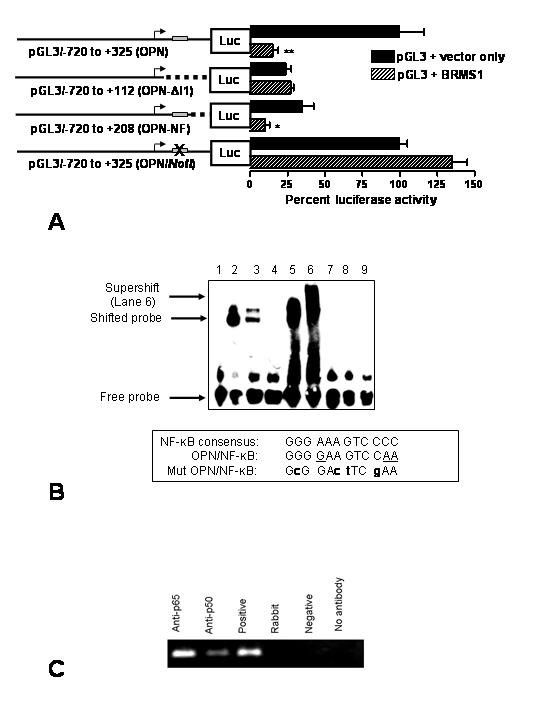
**A**. The OPN promoter lacking the NF-κB site is relieved for BRMS1 suppression. The grey box represents the predicted NF-κB site. The dotted line indicates the region deleted in the construct. OPN-ΔI1 does not have the NF-κB-binding site and is not suppressed by BRMS1. OPN/*NotI*, in which the NF-κB site is abolished by inserting a *NotI *site in its place, is refractory to suppression by BRMS1. Data is expressed as Relative luciferase activity of control, where control (pGL3-OPN) is 100%. The data shown represents more than three independent experiments in triplicate. * indicates significant suppression, p value < 0.05 and ** indicates p < 0.01 compared to respective controls. **B**. Recombinant p65 and p50 bind to and retard the mobility of the predicted NF-κB site from the promoter of OPN. Lane 1: Probe bearing the NF-κB site from OPN promoter; Lane 2: Probe + recombinant p50; Lane 3: Probe + nuclear extract of MDA-MB-435; Lane 4: Probe + unlabeled 'cold' consensus probe + Nuclear extract of MDA-MB-435; Lane 5: Probe + recombinant p65; Lane 6: Probe + recombinant p65 + anti-p65 antibody; Lane 7: Mutant probe; Lane 8: Mutant Probe + recombinant p65; Lane 9: Mutant Probe + recombinant p50. The box gives the sequence comparison of the OPN/NF-κB with the consensus NF-κB-binding site. The underscored bases in OPN/NF-κB represent variation from the consensus. Mutant OPN/NF-κB (Mut OPN/NF-κB) is also shown with the mutations represented in bold lower case. **C**. NF-κB subunits bind to the OPN promoter *in vivo*. MDA-MB-435 cells were fixed with formaldehyde, lysed, and then sonicated. *In vivo *cross-linked chromatin was then precipitated independently using p65 (Anti-p65), p50 (Anti-p50), normal rabbit IgG (Rabbit), no antibody, or Positive and Negative kit control antibodies (Active Motif). The recovered immunoprecipitated DNA was then used for PCR with primers specific for the OPN/NF-κB-containing promoter segment. A 151 bp product corresponding to a region 1575 bp upstream of the OPN/NF-kB site (that lacks a predicted NF-κB site [12]) was absent in a control PCR following ChIP Primers: 5'-TTCCCCCTACCAAATGTTCA-3' and 5'-TGCTGCAAAAGTAATTGTGGTT-3'.

Intron 1 of OPN has been implicated in OPN regulation [11]. Hence, we constructed OPN-ΔI1 which lacked +113 to +325 region, downstream of the transcription start site. This construct was relieved of BRMS1 repression (Fig. 2A). Analysis of this region using Transfac [12] showed a putative NF-κB site at the +124 to +136 position. A deletion of the OPN promoter, OPN-NF (lacking +209 to +325), which has an intact NF-κB site, remained sensitive to suppression by BRMS1. This suggests that BRMS1 may function *via *this putative NF-κB site to suppress OPN. To confirm this, we created OPN/*NotI*, in which the NF-κB site is abolished by inserting a *NotI *site in its place. OPN/*NotI *is refractory to suppression by BRMS1 (Fig. 2A), thereby substantiating the observations made earlier.

Of note, the two deletion constructs (OPN-NF and OPN-ΔI1) have overall lower promoter activity. This is not unexpected since deletions in this region of OPN promoter have previously been reported to have such an effect [11].

### Recombinant p65 and p50 bind to and retard the mobility of the predicted NF-κB binding site from the OPN promoter

The predicted OPN/NF-κB site has 9 conserved and 3 non-conserved bases as compared to a consensus NF-κB binding site (Figure 2B). Hence, to investigate whether this site is recognized by the prototypical members of the NF-κB complex (p65 and p50), we performed an EMSA using double-stranded oligos coding for OPN/NF-κB as well as mutant OPN/NF-κB. As seen in lanes 2 and 5, we observed a shift in probe with recombinant human p50 and p65, respectively (Fig. 2B). An anti-p65 antibody caused a supershift of the probe when incubated with recombinant p65 (Lane 6). The gel mobility of the probe was also retarded by the nuclear extract of 435 cells (Lane 3), which was completely out-competed by an unlabeled ('cold') consensus NF-κB probe (Lane 4). Notably, 435 has constitutively active nuclear NF-κB (p65 and p50) [13]. Hence, this shift confirms that the resident members of NF-κB complex from 435 cells recognize the OPN/NF-κB *cis *element. The mutant OPN/NF-κB probe does not show a shift with p65 and p50 (Lanes 8 and 9).

### NF-κB subunits bind to the OPN promoter *in vivo *in MDA-MB-435

By ChIP assays using anti-p65 and -p50 in MDA-MB-435 cells, we were able to amplify the 201 bp target region from the OPN promoter by PCR (Fig. 2C). The intensity of the band obtained by ChIP using the p50 antibody was consistently lower in multiple repeats. This could be due to weaker immunoprecipitations by the p50 antibody or weaker interaction of p50 with OPN/NF-κB as compared to that of p65. A control PCR designed to amplify a 151 bp region (that lacks any homology to NF-κB site), 1575 bp upstream of the OPN/NF-κB site did not yield a product following ChIP using the p65 or p50 antibody (data not shown). Thus, ChIP specifically pulled down the NF-κB-binding site from the OPN promoter.

### OPN/NF-κB grafting renders SV40 promoter sensitive to BRMS1 suppression

**Figure 3:**
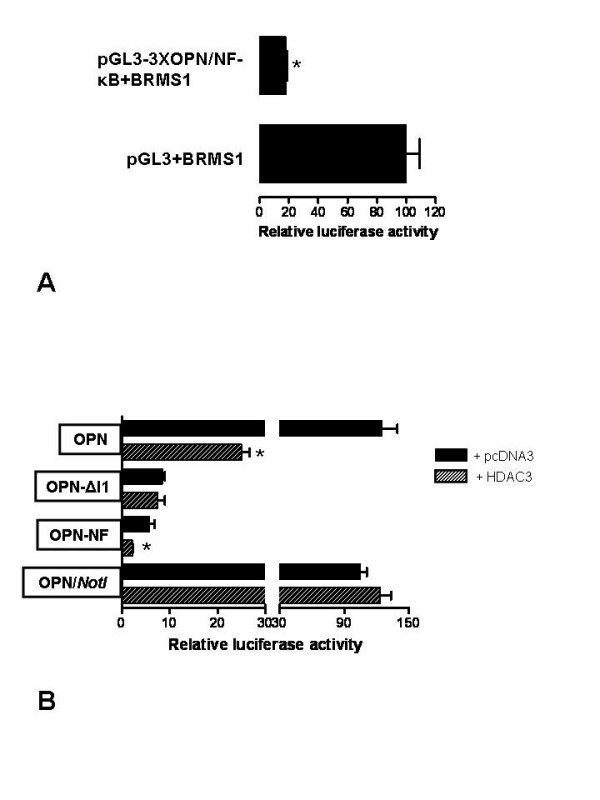
**A**. BRMS1 downregulates OPN *via *the NF-κB site. A reporter plasmid bearing three NF-κB sites from the OPN promoter, pGL3-3XOPN/NF-κB, upstream of the SV40 promoter or pGL3-control was co-transfected with pCMV-mycBRMS1 into COS-7 cells. Luciferase activity from this reporter construct was measured. The experiment was repeated thrice in triplicate.* indicates significant suppression compared with respective controls (p < 0.05). **B**. The OPN promoter lacking an intact NF-κB site is relieved for suppression by HDAC3. The OPN promoter construct, pGL3-OPN, and its deletions, OPN-NF (retains the NF-κB-binding site) and OPN-ΔI1 (lacks the NF-κB-binding site) and the OPN/*NotI *construct (OPN/NF-κB site is replaced with a *NotI *site) were co-transfected with pcDNA3 or pcDNA3-FLAG-HDAC3 in COS-7 cells and monitored for the effect of HDAC3 on the promoter activity of OPN. The results shown represent the experiment done twice in triplicate. * indicates significant suppression compared with respective controls (p < 0.05).

If the OPN/NF-κB site is recognized by BRMS1, grafting it onto another promoter should render that promoter sensitive to BRMS1 repression. We grafted three tandem repeats of OPN/NF-κB (3XOPN/NF-κB) upstream of the SV40 promoter in pGL3-Control vector to generate pGL3-3XOPN/NF-κB. The luciferase reporter assays showed that pGL3-3XOPN/NF-κB is significantly (p < 0.01) repressed by BRMS1 (Fig. 3A). This observation implies that the OPN/NF-κB site is sensitive to BRMS1.

### The OPN promoter construct lacking NF-κB is relieved for suppression by HDAC3

HDAC3 is reported to regulate the acetylation status and transcriptional activity of p65 [14]. Therefore, we evaluated the luciferase activity of full length OPN promoter (pGL3-OPN), the deletion of the OPN promoter with intact NF-κB site (OPN-NF) and the OPN promoter with a deletion of NF-κB site (OPN-ΔI1), in the presence and absence of HDAC3. We observed that the OPN and OPN-NF promoter activity is repressed by HDAC3 (Fig. 3B). Interestingly, the deletion of the OPN promoter (OPN-ΔI1) that was refractory to BRMS1-mediated suppression was also not suppressed by HDAC3. This was also confirmed with the OPN/*NotI *construct (in which the NF-κB site is abolished by inserting a *NotI *site in its place, without changing the length of the construct), which also is refractory to suppression by HDAC3. This suggested that HDAC3 represses OPN through the same *cis*-site as BRMS1.

### BRMS1 associates with HDAC3

**Figure 4:**
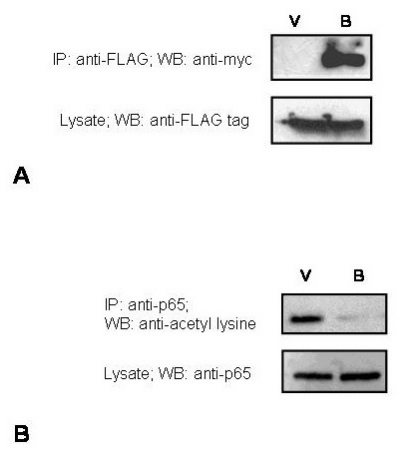
**A**. BRMS1 co-immunoprecipitates HDAC3. FLAG-HDAC3 was co-transfected into COS-7 cells with either pCMV-myc (V) or pCMV-myc-BRMS1(B) using Lipofectamine 2000 (Invitrogen). The cell lysate was immunoprecipitated with 1 μg anti-FLAG Ab (Sigma). The precipitated proteins were resolved by SDS-PAGE and immunoblotted with 1:500 dilution anti-myc Ab (BD Clontech). Independently the cell lysate was immunoblotted with anti-FLAG antibody to confirm expression of FLAG-HDAC3. **B**. BRMS1 reduces acetylation of p65. MDA-MB-435 cells were transfected with pCMV-myc (V) or pCMV-myc-BRMS1 (B) and immunoprecipitated with anti-p65 antibody. The immunoprecipitate was resolved on SDS-PAGE, transferred to PVDF membrane and immunoblotted with anti-acetyl lysine antibody. The lysate shows that the levels of p65 are not altered by BRMS1.

We co-transfected COS-7 cells with FLAG-HDAC3 and myc-BRMS1 expressing plasmids. We were able to co-immunoprecipitate BRMS1 from the lysate using anti-FLAG antibody (Fig. 4A). This observation corroborates with the recently published literature showing that BRMS1 associates with Class I and II HDAC members [15].

### BRMS1 reduces acetylation of p65

MDA-MB-435 has a significant amount of HDAC3. However, it shows constitutive NF-κB with acetylated p65. Since we know from our previous work [7] that these cells do not have significant levels of BRMS1, we tested if re-expressing BRMS1 could influence the acetylation status of p65 in these cells. We immunoprecipitated p65 from 435 cells with and without BRMS1 and immunoblotted with anti-acetyl lysine antibody. We saw that in 435/BRMS1 cells, the otherwise constitutively active (acetylated) p65 had reduced acetylation despite having levels of p65 comparable with the control 435 cells (Fig. 4B).

## Discussion

BRMS1 has been implicated in several molecular events such as restoration of gap-junctional intracellular communication [16] and phosphoinositide signaling [17]. Cicek *et al *[18] have demonstrated that BRMS1 expression leads to the inhibition of IκBα phosphorylation and degradation and subsequently to a reduction of p65 and p50 nuclear translocation. Very recently, Liu *et al *[19] have demonstrated involvement of BRMS1 in mediating acetylation of NF-κB. Our study has demonstrated a known metastasis promoting gene, OPN, to be directly regulated by BRMS1, a metastasis suppressor gene. We observe consistent suppression of OPN by BRMS1 at the RNA and protein level, corroborating our reporter assays using the OPN promoter which show suppression by BRMS1. Further investigations demonstrate that the site responsible for BRMS1-mediated repression of OPN matches closely to an NF-κB consensus site. Interestingly, this site is located after the transcription start site, within intron 1 of OPN. It is known that intron 1 of the OPN gene contains crucial regulatory elements that can influence gene expression [11]. NF-κB is also reported to regulate several genes through *cis*-acting elements located in the first intron [20,21]. There are several NF-κB family members that participate in the formation of different homo- or heterodimers with various degrees of target gene specificity [22]. We have analyzed the prototypical NF-κB complex, which is a heterodimer of p50 and p65 (RelA). Gel shift assays using purified human p65 and p50 confirmed a direct recognition of the OPN/NF-κB site by the NF-κB complex members. The ChIP assay demonstrated that the OPN/NF-κB site is indeed functional *in vivo*. The studies with mutated OPN/NF-κB (in EMSA) as well as a 'grafted' NF-κB site support these findings. More interestingly, we observed that HDAC3 controls the OPN expression through the OPN/NF-κB site. Co-immunoprecipitation studies indicate that BRMS1 is capable of interacting with HDAC3. It is known that HDAC3 deacetylates p65 resulting in decreased activity of NF-κB [14]. Our studies showed that restoration of BRMS1 expression in MDA-MB-435 cell line, which has constitutively acetylated p65 despite significant levels of HDAC3, leads to deacetylation of p65. This results in the inactivation of NF-κB, which is reflected in decreased expression of the target gene, OPN. It is possible that the BRMS1-mediated reduction of OPN expression is due to either reduction in p65 trans-activation or due to decreased binding of NF-κB to the OPN/NF-κB site.

BRMS1 is a known member of the mSin3-HDAC transcription co-repressor complex [10]. Thus, it is expected to interact with HDAC 1 and 2. Since HDAC3 is not a known member of the mSin3-HDAC complex, our work presented here implies the involvement of BRMS1 in other HDAC-containing complex(es).

OPN is an important chemokine, promoter of tumor progression, and indicator of poor prognosis in a variety of cancers [5]. Work by Renault *et al *has implicated NF-κB regulation of OPN [23]. The NF-κB pathway is one of the major signaling pathways responsible for cancer cell invasion and as targets for cancer therapy. Constitutive activation of NF-κB, such as in MDA-MB-435, is observed during progression of breast cancer to hormone-independent growth [14]. Our findings suggest that BRMS1 is able to deactivate this NF-κB by deacetylation of p65 possibly by its association with HDAC3.

## Conclusion

BRMS1 has been shown to suppress metastasis of breast cancer and melanoma in animal model studies. Also, BRMS1 is a member of mSin3-HDAC transcription co-repressor complex. Our work demonstrates that expression of osteopontin, a tumor-metastasis promoting protein, is regulated by BRMS1. We also demonstrate that this regulation is brought about by abolishing NF-κB activation of OPN promoter. We also show that the OPN promoter is sensitive to repression by HDAC3 *via *the OPN/NF-κB site. Our studies imply that BRMS1 likely suppresses OPN by abrogating activation of NF-κB. Thus, downregulation of OPN may be one of the mechanisms of metastasis suppression by BRMS1.

## Materials and methods

### Cell lines

MDA-MB-435 (referred to as 435), a gift from Dr. Janet Price (University of Texas-M. D. Anderson Cancer Center) is a human metastatic breast carcinoma cell line [24]. (Note: There is a debate about the origin of this cell line (melanoma or breast cancer) [25-27], however BRMS1 has been shown to suppress metastasis of both these cancers. Hence we used this cell line as it is the best model for the regulation study of OPN. BRMS1-transfected 435 (435/BRMS1) were generated by us and the culture conditions have been described previously [7]. For all functional and biological assays, cells with >95% viability were used at 70–90% confluence. All the lines were routinely checked and found negative for *Mycoplasma spp*. using the TaKaRa Mycoplasma detection kit (TaKaRa Bio, Otsu, Shiga, Japan).

### Plasmids and transfections

pCMV-myc-BRMS1 was constructed by cloning the BRMS1 ORF in pCMV-myc (BD-Clontech, CA, Palo Alto, USA). The human OPN promoter construct was a gift from Dr. Iizuka, Hokkaido University, Japan [28]. PCR-generated deletions of the OPN promoter (indicated in Fig.2A) were cloned in the pGL3-basic vector (Promega, Madison, WI, USA).  A 3XOPN/NF-κB construct, pGL3-3XOPN/NF-κB, was made by cloning commercially synthesized oligomers bearing the OPN/NF-κB site repeated three times in tandem into pGL3-Control vector (Promega). The NF-κB site in the OPN promoter was disrupted and replaced with a *Not*I site (OPN/*NotI*) using the oligos 5'-GATCGATCGTGCGGCCGCAAATTCTAAGGAAAAATATTTTTAATTGTAATGCTG-3' and 5'-GATCGATCGTGCGGCCGCATGTTTTTCAGCTGAATGCACAAC-3' with pGL3-OPN as a template for inside-out PCR.

The pcDNA3-FLAG-HDAC3 plasmid was a gift from Dr. Edward Seto, University of South Florida, FL, USA [29].

### Immunoblotting

To determine OPN expression, 4 × 10^6 ^cells were seeded in 5% FBS containing medium. After 24 hours, the medium was replaced with serum-free medium and assayed 24 hours later for OPN expression. The cell-free medium was resolved using a 12.5% SDS-PAGE. Proteins were transferred to a PVDF membrane and probed with the anti-human OPN mouse monoclonal antibody [30] (1:1000) followed by secondary antibody conjugated to horseradish peroxidase (Amersham Biosciences, Piscataway, NJ, USA) and detected using chemiluminescence (Amersham BioSciences). OPN is seen at ~55–65 kDa. Cell lysates were prepared as described previously [10] and 30 μg was resolved using a 12.5% SDS-PAGE and immunoblotted with the respective primary antibody followed by detection using Supersignal West Dura (Pierce, Rockford, IL, USA). Epitope-tagged BRMS1 expression from 435/BRMS1 was detected using anti-901 epitope tag antibody, described previously by us [7]. Densitometric analysis was performed using the digital densitometry analysis tool of AlphaEase^®^FC image analysis software.

### Immunoprecipitation

COS-7 cells were co-transfected with pCMV-myc-BRMS1 and pcDNA3-FLAG or pcDNA3-FLAG-HDAC3 using Lipofectamine 2000 (Invitrogen, Carlsbad, CA, USA) per the manufacturer's instructions. Transfected cells were lysed and immunoprecipitated with 1 μg anti-FLAG Ab (Sigma) and immunoblotted as described above. The membrane was probed with 1:500 dilution anti-myc Ab (BD Clontech, Palo Alto, CA, USA). For analysis of the acetylation status of p65, 300 μM Trichostatin A (Calbiochem, EMD Biosciences, La Jolla, CA) was added to the lysis buffer to halt HDAC activity released upon cell lysis. The cell lysate was immunoprecipitated with the anti-p65 antibody (Santa Cruz) followed by the rabbit anti-acetyl lysine antibody (1:1000) (Chemicon, Temecula, CA, USA) for immunodetection.

### Luciferase reporter assays

COS-7 cells were transfected using Lipofectamine 2000 (Invitrogen) per the manufacturer's instructions. Total protein was harvested (Luciferase assay kit, Promega) and the luciferase activity measured using a Turner 20/20 luminometer (Turner Biosystems, Sunnyvale, CA, USA). The luciferase reading was normalized to the total protein concentration. Data is expressed as Relative luciferase activity, where control is 100%.

### Electrophoretic mobility shift assay (EMSA)

Nuclear proteins were isolated from MDA-MB-435 cells grown until approximately 85% confluent in a 100 mm dish using the protocol of Zayzafoon, et al [31]. Binding reactions containing either 10 μg nuclear extract or 100 ng or 66 ng, respectively, of recombinant p65 (Active Motif, Carlsbad CA) or p50 (Promega), 1 μg poly (dI-dC), and 20 fmol biotinylated oligonucleotide probe in buffer (10 mM Tris, 50 mM KCl, 1 mM DTT, 5 mM MgCl_2_) were incubated at room temperature for 30 minutes.

The oligonucleotide probes correspond to the OPN/NF-κB site (5'-GAATTTCATGGGGAAGTCCAAATTCTAAG) or Mut OPN/NF-κB (5'-GAATTTCATGCGGACTTCGAAATTCTAAG).

A consensus NF-κB probe (5'-AGTTGAGGGGACTTTCCCAGGC) served as a specific inhibitor. For antibody supershift, the samples were preincubated with 2 μg of anti-p65 antibody (Santa Cruz). Gel electrophoresis, blotting and development followed the manufacturer's protocol of the Chemiluminescent LightShift Assay Kit (Pierce). Four pmol of the consensus NF-κB probe (5'-AGTTGAGGGGACTTTCCCAGGC) served as a specific cold competitor.

### Chromatin immunoprecipitation

Cells (435) were utilized for chromatin immunoprecipitation using the ChIP-IT kit (Active Motif) as directed by the manufacturer using p65 or p50 antibody. Parallel controls for each experiment included samples with no chromatin, no antibody, normal rabbit IgG (Santa Cruz Biotech), and the kit-provided positive (RNA polymerase II) and negative control antibodies. After elution and purification, the recovered immunoprecipitated DNA samples were used for PCR (Platinum Taq polymerase; Invitrogen) using primers [5'-CAGTTGCAGCCTTCTCAGC-3' (forward) and 5'-CCTTTGTTCCACAGGAGACC-3' (reverse)] to amplify a 201 bp segment of the OPN promoter containing the NF-κB site. PCR products were analyzed by agarose gel electrophoresis.

The specificity of the pull-down was confirmed by amplifying a region 1575 bp upstream from the PCR product containing the NF-kB site tested. The primers used were 5'-TTCCCCCTACCAAATGTTCA-3' and 5'-TGCTGCAAAAGTAATTGTGGTT-3'. The PCR generates a 151 bp product. This segment lacks a predicted NF-κB site [12].

### Statistical analysis

Statistical analysis was done using the unpaired one-tailed Student's t-test (Graphpad Prism, San Diego, CA, USA).

## Abbreviations

BRMS1 Breast cancer Metastasis Suppressor 1

ChIP Chromatin Immunoprecipitation

DMEM-F12 Dulbecco's Modified Eagle's Medium:F12 medium (1:1)

EMSA Electrophoretic mobility shift assay

HDAC Histone deacetylase

ORF Open reading frame

OPN Osteopontin

PCR Polymerase chain reaction

PVDF Polyvinylidine fluoride
